# Relative effects of mammal herbivory and plant spacing on seedling recruitment following fire and mining

**DOI:** 10.1186/1472-6785-7-13

**Published:** 2007-10-29

**Authors:** Michael H Parsons, Christine M Rafferty, Byron B Lamont, Kenneth Dods, Meredith M Fairbanks

**Affiliations:** 1 Centre for Ecosystem Diversity and Dynamics (CEDD) in the Department of Environmental Biology, Curtin University of Technology, PO Box U1987, Perth, WA, Australia; 2 Whiteman Park, Lord Street, Whiteman, WA 6068, Australia; 3 Chemistry Centre (WA), 125 Hay Street, East Perth, WA 6004, Australia; 4 Department of Agriculture, Locked Bag 4, Bentley, WA 6983, Australia

## Abstract

**Background:**

There is much debate concerning which ecological constraints are the most limiting factors to seedling recruitment in disturbed communities. We provide the first comparison between selective herbivory and plant competition effects among two post-mined forest ecosystems (primary succession) and one post-fire woodland ecosystem (secondary succession). Animal exclosure assessments of nine common species across eight sites were performed for comparison within three locations separated by up to 200 km. Additionally, we asked whether pre-browsed plants differed in nutrient content between or within species in the separate systems.

**Results:**

Among the nine common species, seven of these were affected by mammal herbivory while five shared a similar vulnerability to predation regardless of system. One species was limited by competition (planting density). There was a strong linear correlation between herbivore selectivity (% browsed) and impact (biomass loss) on the fertilized minesites, but not post-fire sites. Phosphorus and potassium were higher for most species in the post-mined system. Principal components analyses revealed that nutrients in shortest supply may be the most likely components of selection within each system. Among all locations, species with highest levels of phosphorus, ADF and leaf water content were often favoured, while high tannins and nitrogen content were generally selected against.

**Conclusion:**

Herbivory, rather than seedling competition, was the limiting factor for plant performance among post-fire and post-mined reclamation areas. The post-fire seedlings were smaller and more water and nutrient limited, nevertheless browsing prevalence was equivalent at all locations with nearly all seedlings predated. Kangaroo density in the post-fire community declined from the beginning of the experiment, while numbers in the post-mined revegetation increased fourfold within one year. Differences in water and nutrient availability may explain why herbivores are more likely to be attracted to post-mined communities.

## Background

Herbivory and plant competition are the primary constraints to seedling recruitment in recently disturbed ecosystems. It is often debated whether herbivory or competition has a greater impact on seedling recruitment [1,2]; interactions between the two processes are a common experimental problem, making system assessments difficult [3]. For example, herbivores may browse plants at higher density (closely spaced) as predicted by optimal foraging theories [4], or may select plants that are rare according to their nutritional needs [5,6]. For these reasons, such interacting processes are best examined together [2].

Early competition in Mediterranean-climate disturbed communities is for water (seasonal drought) and, to a lesser extent, nutrients (mined sites are fertilized and ash is available after fire), but not for light, as the ecosystems are open. The western grey kangaroo (*Macropus fuliginosus*) is the most common native herbivore that impacts reclamation efforts in Australia. The introduced European rabbit (*Oryctolagus cuniculus*) is also abundant throughout the continent.

Post-mining reclamation has been referred to as the ideal case study for forest regeneration studies starting at 'point zero or terra nova' [7]. Provision of roads and tracks in mined areas exposes undergrowth to browsers [8] and makes areas easily negotiable [9]. The newly sown seedlings are moist and more palatable than older plants [10], and are usually fertilised. These nutrient enriched areas may be more prone to vertebrate browsing by increasing plant vigor [11], and consequently, may attract herbivores from farmland into newly restored areas [12].

Secondary succession differs from primary succession in that soil is already pre-formed (relatively undisturbed) and ready for use by plants. Additionally, the environment has already been used by the plant species under consideration; they are simply cycling back into the system after the disturbance. In primary succession the species are typically new to the environment. There is still disagreement regarding the extent to which kangaroos are attracted to areas of secondary succession.

Southwell suggested Aborigines used knowledge of newly burned areas as a tool to hunt kangaroos [13]. The practice of 'fire stick farming' was common to attract native fauna into vegetation mosaics [14]. Likewise, Denny [15] witnessed a fourfold increase in kangaroo density the first year after burns. In contrast, Caughley [16] saw no increase in kangaroo numbers following burns.

### Components of selection vary by habitat

Nutritive status and palatability may differ between plant species or locations, which may influence total browsing patterns. Components of selection for generalist herbivores typically include essential nutrients in short supply, including salts, phosphorus and protein [17], and may be balanced by the presence of toxins such as tannins and other plant secondary metabolites. Other factors such as plant architecture [18] may play a role in selection. Kangaroos often show a preference for grasses, switching to this food source whenever it is available. Positive selection for grasses has been attributed to their greater nutrient content [12], especially high nitrogen levels [19]. Yet macropod kidneys are efficient at recycling nitrogen [20] and Parsons et al. [21] demonstrated that nitrogen was not positively correlated with western grey kangaroo diet selection (percent of plants browsed). However, they acknowledge that total plant biomass consumed was correlated with high nitrogen content. Kangaroos in both natural and post-mining systems are known to prefer grasslike species [19,22], though a myriad of plant species consumed form a more varied diet than previously thought [5,23]. It is not known if the same species are impacted in similar ways in differing ecosystems.

Our aim was to compare levels of herbivory and competition (close-spacing) on young plants of shared species in three ecosystems, one post-fire and two post-mined. We then determined if plant chemical components correlated with the extent of animal impact were the same in the three system-types. To our knowledge, ours is the first time any direct comparison has been performed between post-fire and post-mined systems.

We asked:

1. Is mammal herbivory more important than plant competition (density) in all three ecosystems?

2. Are herbivory and plant competition impacts similar between post-mined and post-fire systems?

3. Is impact (biomass loss) a function of the level of herbivore activity (percent eaten)?

4. Are preferred species impacted equally in each system and do they have similar nutrient and/or toxin levels?

5. Does herbivore visitation increase following post-fire and mining reclamation?

### Study Areas

We focus our study on two disturbed ecotypes – one located in banksia woodland (post-fire) and two located in eucalypt forest (post-mining). Descriptions of all locations are provided in Table 1. The region has a Mediterranean-type climate with cool, wet winters and hot, dry summers. All areas are low in phosphorus due to long periods of leaching weathered materials from parent rock and fixation to iron oxides.

**Table 1:** Table of attributes for 3 post-disturbance locations. Max/min temperature is monthly. 1 = western grey kangaroo, 2 = rabbit, 3 = western brush wallaby

Ecosystem	Site	Location relative to Perth	Major species prior to disturbance	Landform	Soil type	Rainfall (mm)	Max/min temp (°C)	Type of disturbance	Primary herbivores
Banksia woodland	Whiteman Park	18 km NE	*Banksia attenuata*, *B. menziesii*, *B. ilicifolia*, *B. littoralis*, *Acacia saligna, Allocasuarina fraseriana*	Coastal dunes of quartz origin	Deep sands	700	32.5/8	Fire	1,2,3
Moist jarrah forest	Huntly	100 km S	*Eucalyptus marginata*, *Corymbia calophylla, B. grandis*, *A. fraseriana*, *Persoonia longifolia*	Plateau of granite origin	Laterite over clay loam	1200	28/5	Bauxite mining	1,2,3
Dry jarrah forest	Boddington	180 km SE	*E. marginata*, *C. calophylla, B. grandis*, *A. fraseriana*	Plateau of granite origin	Laterite over clay loam,	800	30/5	Bauxite mining	1,3

The post-fire assessment was carried out at Whiteman Park, a conservation and leisure reserve of 3 600 hectares, located 18 km NE of Perth. Fire affected much of the conservation area at Whiteman Park on 14 February 2001, providing an ideal opportunity to observe post-fire herbivory activity by the primary native vertebrate herbivores in the area. The post-mining assessments were carried out at Alcoa World Alumina Australia's Huntly mine (Huntly; 100 km S of Perth) and Worsley Alumina Pty Ltd's Boddington bauxite mine (BBM; 180 km SE of Perth). Both mines are located in the Darling Range, southwestern Australia. The mine pits range in size from one to ten hectares and are surrounded by relatively intact native forest. The rehabilitation process involves reshaping the mine pit, return of the topsoil and deep ripping to relieve compaction. Seeds of local plants are spread throughout the rehabilitated mine pit and nursery grown seedlings are planted.

## Methods

The aim of our study was to demonstrate, explain and compare selective browsing and plant competition effects among common species that establish following major environmental disturbance in disparate ecosystems. We aimed to conduct our tests on a broad (subregional) scale and with extended duration (up to 12 months). Between May and July 2001(autumn/winter), pairs of exclosures were erected at three sites each at three locations: Whiteman Park (31°48'09.7"S, 115°56'17.1"E); Huntly mine (116°08'E, 32°34' S) and BBM (116°28' E, 32°58' S). All mine sites were in separate pits that were routinely rehabilitated in summer/autumn 2001. Plant species selected for the experimental study had a wide range of physical and chemical morphologies. Only plants indigenous to the study sites were selected; this included nineteen species at Whiteman Park and twenty-seven on mine sites. This study focuses on the nine common species shown in Table 2.

**Table 2:** Species in common at the 3 locations. * = grasslike as seedlings

Species (family)
1. *Acacia alata *(Mimosaceae)*
2. *Acacia pulchella *(Mimosaceae)
3. *Allocasuarina fraseriana *(Casuarinaceae)*
4. *Cyathochaeta avenacea *(Cyperaceae)*
5. *Mirbelia dilatata *(Fabaceae)
6. *Austrodanthonia caespitosa *(Poaceae)*
7. *Sphaerolobium vimineum *(Fabaceae)
8. *Xanthorrhoea gracilis *(Xanthorrhoeaceae)*
9. *Xanthorrhoea preissii *(Xanthorrhoeaceae)*

Exclosure pairs, one permanent and one temporary, measured 25 m × 25 m, with a buffer strip (25 m × 5 m) left between each. Fences were constructed from Ringlock^® ^fencing wire attached at 5 m intervals to 2.5 m steel star pickets. Fine chicken mesh was attached to the lower third of each fence and dug in 0.5 m. The experimental area was divided into cells of 1.3 × 1.3 m. Nine plants of each study species were planted in each cell. Four replicates were planted as low-density plots, with the distance between adjacent plants at 0.5 m. A further four replicates were planted as high-density plots, with the distance between plants at 0.1 m. Plants were randomly allocated to the cells in each exclosure, with the provision that high and low density plots alternated throughout the grid. Prior to planting at Whiteman Park, the surface of all sites was dug over to remove naturally resprouting plants. Temporary exclosures were removed from November 2001 to January 2002 (summer), after plants had established. These dates were selected to commence the period when herbivore damage was thought to be maximal (H. Gratte, Whiteman Park, pers. comm.), with most annual grasses diminished and little new growth available during mid-summer in the area.

Two leaves (youngest fully extended) were collected from each of ten plants of each species, and leaf mass: area and thickness were determined as described by Witkowski and Lamont [24]. Correction was made to the formula for leaf mass: area of needle-leaved species by dividing rather than multiplying the standard formula by 0.7854. Leaf area was measured using the Dias system (Delta-T Devices, Cambridge, England). Plant height was calculated for each species by taking the average of five plants at each site in each exclosure.

Harvest time was selected to provide plants with sufficient time to recover from bouts of herbivory while still showing its long-term effects. Ten months to one year after removal of temporary exclosures, plants were clipped at ground level, wrapped in dampened paper towel and sealed in freezer bags. One plant per species-replicate (group of nine) was placed in cooler boxes and refrigerated until weighing to give a fresh biomass value per plant. Plants were dried in brown paper bags at 60°C for 48 hours in an air-forced oven. Plant weights at each plot were averaged to give a dry plant weight per plot value. Average water content (%) was calculated for each species as the difference between wet and dry weights. The number of macropod scat and rabbit latrines was recorded within 1 m of all individual seedlings at each site. Scat counts were performed monthly at Whiteman Park, and before and after the exposure period on the mine sites.

### Chemistry

One plant from each group of nine replicates was selected for chemical analysis. Harvested shoots were milled through a crossbeater mill to a uniform particle size of <1 mm. A sub-sample was then taken and assayed for moisture using a recommended two-stage method of moisture determination for organic material [25]. All results were calculated on a dry basis. Total nitrogen was determined by the Berthelot colorimetric method [26] after sulfuric/salicylic acid/hydrogen peroxide digestion [27]. Crude protein was determined by multiplying the nitrogen content by 6.25 [28]. The plant material was simmered in an acidic detergent solution for 1 h and then filtered on a coarse sintered glass crucible. The weight of the dry residue gave a measure of the acid detergent fiber (ADF), after allowing for ash content according to the method 973.18 [29]. Plant material was extracted in 70% acetone. An aliquot of the supernatant was then taken and reacted with Folin Ciocalteu reagent and sodium carbonate. To obtain total tannins, polyvinyl pyrilidine (PVP) was then added to the total polyphenolic extract. PVP precipitatable phenolics (tannins) were then determined by difference in the absence of PVP, and expressed as tannic acid equivalents [30]. Macro elements were analysed using inductively coupled plasma atomic emission spectrometry after acid digestion with nitric and perchloric acids [31].

### Statistics

We used shoot dry mass as our response variable. We recorded values from low and high density plots during the harvest period. ANOVA with interactions, Minitab v14, Minitab Inc., State College PA, U.S.A, was applied to herbivore and density effects for individual species at both mine locations (three-way: location × exclosure × density) with sites as replicates. As no significant interactions were observed between mine locations, we combined mine site data and compared to these to the post fire location using the same model (three-way: location × exclosure × density). We then performed a pairwise comparison of means for exclosure and density effects for each system. We set our risk level at α = 0.05.

Numbers of plants per species showing signs of herbivore damage were converted to percentage values for use in multivariate analysis after arc sine transformation. Initial height and initial shoot mass were recorded as predictors along with nutrient analysis, area of the youngest fully extended leaf (YFEL), and leaf mass: area (LMA). Two-way ANOVA was used to test effects of location × species (with interactions) for all nutrients. Pairwise comparisons were performed using Estimated Marginal (EM) means to compare nutrient concentrations within and between sites. All values were analysed using Principal Components Analysis (PCA) in SYN-TAX 2000, Exeter Inc., Setauket, NY, U.S.A [32]. Pearson's correlation coefficient was then used to determine whether there was a linear relation between plant selection (% plants browsed) and impact [log (1 - *P *value)] at each location [21]. *P *is the significance level of the difference between shoot mass inside and outside the exclosures. As a way of standardizing the results, *P *is subtracted from 1 to give an index of herbivore impact. The difference in scats between pre and post-harvest were compared by one-way ANOVA using SPSS version 11.5 (SPSS Inc., Chicago, IL, U.S.A.). Scheffé s post-hoc test was used to compare scat counts between locations.

## Results

More than 70% of seedlings were browsed for eight of nine species across eight of the nine sites among the three locations. One site at BBM was omitted from analysis as it was isolated from all herbivores due to heavy mining in the area. *Acacia pulchella *was the least browsed plant. There were no significant differences between overall percentage of plants browsed between locations (ANOVA 2,24; *F *= 0.55, *P *= 0.583: Table 3). Only one interaction was observed in the comparisons between minesites (location χ density *Mirbelia dilatata*); thus we combined data for both minesites for comparison to the post-fire location (Table 4). Overall, seven of nine species showed significant exclosure effects. No density effects were observed at this level. Location effects were seen for seven of nine species.

**Table 3:** Table of variables for the 9 common species in the 3 locations including levels of herbivory. N, P, K, and ADF: % dry weight, H_2 _O: % fresh weight, tannins: Merk tannic acid equivalents. Superscript refers to Estimated Marginal Means post-hoc analysis between sites with different letters significantly different at *P *< 0.05, '*a*' refers to lowest value. YFEL = area of the youngest fully extended leaf

Species	Pre fence removal height (cm)	Pre fence removal shoot mass (g)	Leaf area (mm^2^) YFEL	LMA (μg mm^-2^)	H_2_O	N	P	K	Tannins	ADF	Plants eaten (%)
**Whiteman Park**											
											
*Acacia alata*	31.1	0.41	66	72	50.6	1.33^a^	0.04^a^	0.60^a^	5.38^a^	31.7^a^	81
*Acacia pulchella*	48.6	1.97	45	104	37.7	1.63^a^	0.03^a^	0.47^a^	8.35^a^	28.1^a^	42
*Allocasuarina fraseriana*	20.2	0.38	112	249	59.8	1.06^b^	0.05^a^	0.92^a^	9.07^a^	40.4^a^	71
*Cyathochaeta avenacea*	11.2	0.04	101	167	55.8	1.37^a^	0.04^a^	0.74^a^	2.30^a^	38.2^a^	100
*Mirbelia dilatata*	32.4	0.45	108	107	63.1	1.25^a^	0.03^a^	0.51^a^	0.14^a^	39.4^a^	86
*Austrodanthonia caespitosa*	43.6	0.36	85	162	33.1	0.78^a^	0.03^a^	0.41^a^	0.44^a^	39.5^a^	100
*Sphaerolobium vimineum*	15.5	0.09	14	398	58.0	1.18^a^	0.03^a^	0.55^a^	0.59^a^	46.3^a^	100
*Xanthorrhoea gracilis*	10.7	0.04	105	143	58.1	1.00^a^	0.08^a^	0.70^a^	1.79^a^	37.9^a^	98
*Xanthorrhoea preissii*	9.6	0.04	113	166	60.4	0.97^a^	0.08^b^	0.51^a^	1.83^a^	27.7^a^	98
**Huntly Mine**											
											
*Acacia alata*	42.8	1.97	450	188	52.2	1.90^b^	0.10^c^	1.00^b^	4.32^a^	38.3^a^	99
*Acacia pulchella*	71.5	0.89	100	216	51.7	1.91^b^	0.08^b^	0.86^b^	5.35^a^	27.1^a^	57
*Allocasuarina fraseriana*	39.3	0.81	68	390	58.2	1.03^b^	0.04^a^	0.99^a^	8.27^a^	40.6^a^	99
*Cyathochaeta avenacea*	19.2	0.15	16.7	110	66.5	1.74^b^	0.07^b^	0.94^a^	2.10^a^	44.4^a^	100
*Mirbelia dilatata*	40.1	0.59	116.7	157	67.1	1.55^b^	0.07^b^	0.91^b^	0.25^a^	32.9^b^	93
*Austrodanthonia caespitosa*	55.7	1.18	516.7	456	51.6	0.94^a^	0.05^b^	0.68^b^	0.22^a^	39.8^a^	100
*Sphaerolobium vimineum*	19.0	0.29	200	300	70.5	1.47^b^	0.06^b^	1.62^b^	0.56^a^	39.7^a^	100
*Xanthorrhoea gracilis*	24.6	0.45	41.7	40	58.8	1.12^a^	0.11^b^	0.72^a^	1.53^a^	26.8^b^	98
*Xanthorrhoea preissii*	43.9	0.26	29.2	114	62.3	0.87^a^	0.06^a^	0.60^a^	1.84^a^	23.3^a^	99
**Boddington Mine**											
											
*Acacia alata*	47.4	0.38	428.9	100	61.7	1.51^a^	0.07^b^	0.85^b^	6.03^a^	38.1^a^	79
*Acacia pulchella*	68.5	0.44	65.8	85	55.6	1.57^a^	0.05^b^	0.83^b^	5.83^a^	32.2^b^	76
*Allocasuarina fraseriana*	13.4	0.13	61.3	139	62.0	0.79^a^	0.17^b^	0.81^a^	8.81^a^	42.7^a^	100
*Cyathochaeta avenacea*	----	----	----	----	----	-----	-----	-----	-----	-----	-----
*Mirbelia dilatata*	35.6	0.35	164.5	87	72.4	1.68^b^	0.11^c^	1.00^b^	0.38^a^	31.0^b^	84
*Austrodanthonia caespitosa*	47.4	0.92	123.0	105	64.8	0.96^a^	0.07^b^	0.69^b^	0.49^a^	35.3^a^	100
*Sphaerolobium vimineum*	----	----	----	----	----	-----	-----	-----	-----	-----	-----
*Xanthorrhoea gracilis*	9.5	0.05	206.4	72	67.6	0.93^a^	0.13^c^	0.61^a^	1.39^a^	33.8^b^	95
*Xanthorrhoea preissii*	13.5	0.08	227.0	78	51.8	0.86^a^	0.11^b^	0.58^a^	1.96^a^	26.8^a^	98

**Table 4:** Within-species comparison: mean biomass with interactions for inside/out, low/high density for Whiteman (N = 3) and mines (N = 5) by 3-way ANOVA (site × density × exclosure), exclosure one-tailed, competition and interactions two-tailed

**Species**	Location	Exclosure	Density	Loc χ Excl	Loc χ Dens	Excl χ Dens	Loc χ Excl χ Dens
*Acacia alata*	**0.002**	0.155	0.346	0.378	0.862	0.832	0.490
*Acacia pulchella*	**0.001**	0.062	0.111	0.180	0.276	0.332	0.604
*Allocasuarina fraseriana*	0.093	**0.001**	0.883	0.072	0.751	0.876	0.526
*Cyathochaeta avenacea*	**0.002**	**0.000**	0.242	0.038	0.140	0.773	0.964
*Mirbelia dilatata*	**0.000**	**0.000**	0.678	0.783	0.482	0.801	0.551
*Austrodanthonia caespitosa*	**0.000**	**0.000**	0.911	0.335	0.123	0.126	0.922
*Sphaerolobium vimineum*	0.203	**0.007**	0.613	0.994	0.639	0.479	0.791
*Xanthorrhoea gracilis*	**0.000**	**0.000**	0.348	0.051	0.178	0.972	0.646
*Xanthorrhoea preissii*	**0.000**	**0.001**	0.477	0.035	0.794	0.566	0.768

Eight of the nine species on mine sites were far larger than same species at Whiteman Park. Pairwise comparison (Table 5) showed six post-fire species were significantly affected by herbivory. One density effect was observed; *Cyathochaeta avenacea *had a slightly reduced shoot mass at high density. Among the post-mined areas, seven species showed herbivory effects while we reserve judgement on one species (*P *= 0.062). Six of these species performed similarly between the three locations. *Acacia pulchella *was the only species to resist herbivory throughout all systems. No plants showed density effects at either mine.

**Table 5:** Post-fire vs Post-mine analysis (3-way ANOVA) for location × exclosure × density with all interactions.*P *values are bold where significant. * = not planted at BBM. # = site effect, & = density × exclosure effect. *P *values are bold where significant

**Whiteman Park**	Biomass (g)					
	Exclosure			Density		

Species	Inside	Out	*P*	Low	High	*P*
						
*Acacia alata*	6.06	0.248	**0.010**	3.83	2.47	0.883
*Acacia pulchella*	28.5	11.07	0.201	19.3	20.3	0.205
*Allocasuarina fraseriana*	3.23	0.500	**0.002**	1.77	1.97	0.729
*Cyathochaeta avenacea*#&	0.14	0.001	**0.020**	0.83	0.53	**0.012**
*Mirbelia dilatata*	40.2	3.700	**0.026**	22.4	18.1	0.816
*Austrodanthonia caespitosa*	2.70	0.001	**0.005**	0.70	2.05	0.117
*Sphaerolobium vimineum*	1.33	0.001	**0.042**	0.55	0.75	0.617
*Xanthorrhoea gracilis*	0.01	0.001	0.270	0.006	0.001	0.388
*Xanthorrhoea preissii*	0.02	0.002	0.103	0.003	0.016	0.306

**Mines **(combined)						

*Acacia alata*	21.5	17.6	0.062	11.6	27.5	0.344
*Acacia pulchella#*	110.0	84.4	0.135	119.9	74.5	0.242
*Allocasuarina fraseriana#*	4.48	1.49	**0.011**	3.56	2.81	0.463
Cyathochaeta avenacea*	0.42	0.02	**0.001**	0.26	0.17	0.277
*Mirbelia dilatata*	19.6	2.17	**0.019**	5.93	15.9	0.443
*Austrodanthonia caespitosa*	14.1	0.18	**0.000**	7.56	6.10	0.909
Sphaerolobium vimineum*	1.45	0.07	**0.018**	1.12	0.40	0.277
*Xanthorrhoea gracilis*	0.07	0.02	**0.002**	0.06	0.03	0.060
*Xanthorrhoea preissii*	0.12	0.01	**0.001**	0.07	0.06	0.694

**Figure 1:**
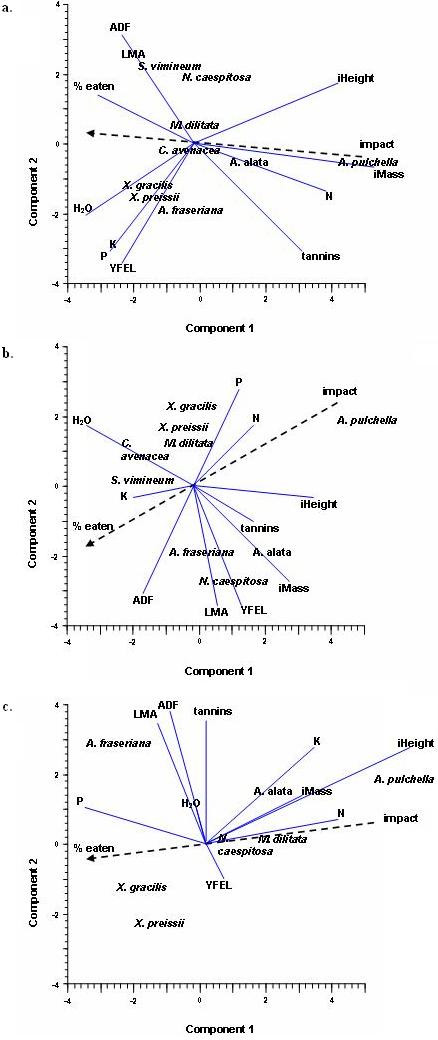
**Macropod raw scat counts at 3 locations, 2002**. First count taken after establishment, final counts taken before harvest. Solid fill represents January-February count, diagonal fill is August-September. Letters refer to Scheffé post-hoc test. Rabbit scats were rare.

The three locations showed significantly different macropod scat counts (ANOVA: *F*_2,9 _= 6.771, *P *= 0.029) while rabbit scat counts were rare and not significantly different between locations (ANOVA: *F*_2,9 _= 2.100, *P *= 0.204; Fig 1.). The Scheffé test revealed kangaroo scat density was similar between Whiteman and BBM sites, and between BBM and Huntly. Number of scats varied between sites and seasons at Whiteman. Macropod scats peaked in early autumn, decreasing in number with time. Scat numbers were uniform between sites and location on the mines, with the numbers increasing fourfold by the second harvest at all mined sites.

Nitrogen, phosphorus, potassium and ADF differed by location (Table 3; *df *= 2; *P *= 0.000 – 0.048). Tannins were not significantly different between locations *P *> 0.1. There were species × location interactions for phosphorus and potassium (*df *= 14, *P *= 0.000 – 0.001). Eight of the nine species at Whiteman Park were lower in phosphorus than the same species on the mines; Huntly values were highest (Table 3). Potassium was also lower at Whiteman Park for seven species; Huntly values were the highest. All species on the mines were more enriched with nitrogen than Whiteman Park; Huntly values were highest. Tannins and ADF were similar by location. *Acacia *species were highest in nitrogen content at all sites and locations, *Austrodanthonia caespitosa *and *Xanthorrhoea *spp. had lowest concentrations, all other nutrient concentrations within locations varied by species (Table 6).

**Table 6:** Chemical analysis for each species by location. Plants harvested and analysed for nitrogen (N), protein, phosphorus (P), Potassium (K), acid digest fibre (ADF) and total tannins (TT). N, protein, P and K are given in % dry matter, ADF as % and TT as % equivalent to tannic acid. Means followed by different letters are significantly different at the 95% confidence level, SE in parenthesis. Superscript refers to Estimated Marginal Means post-hoc analysis within sites with different letters significantly different at *P *< 0.05; ('a' refers to largest value, 'b' is smaller etc.)

	N	P	K	ADF	Tannins
**Whiteman Park**	%db	%db	%db	%	%
*Acacia alata*	1.5 (0.19)^ab^	0.05 (0.01)^b^	0.6 (0.26)^bc^	40.1 (3.35)^b^	5.8 (1.15)^b^
*Acacia pulchella*	1.6 (0.41)^a^	0.03 (0.01)^b^	0.5 (0.12)^c^	28.1 (9.92)^c^	8.3 (0.91)^a^
*Allocasuarina fraseriana*	1.1 (0.21)^c^	0.05 (0.02)^b^	0.9 (0.16)^a^	40.4 (1.17)^b^	9.1 (0.52)^a^
*Cyathochaeta avenacea*	1.4 (0.09)^b^	0.04 (0.01)^b^	0.7 (0.11)^b^	38.2 (1.91)^c^	2.3 (0.26)^c^
*Mirbelia dilatata*	1.3 (0.10)^b^	0.03 (0.01)^b^	0.5 (0.11)^c^	39.4 (1.63)^c^	0.1 (0.08)^e^
*Austrodanthonia caespitosa*	0.8 (0.11)^d^	0.03 (0.01)^b^	0.4 (0.09)^c^	39.5 (0.57)^b^	0.4 (0.36)^d^
*Sphaerolobium vimineum*	1.2 (0.16)^bc^	0.03 (0.01)^b^	0.6 (0.20)^c^	46.3 (1.55)^a^	0.6 (0.31)^d^
*Xanthorrhoea gracilis*^	1.0^c^	0.07^b^	0.7^b^	37.9^b^	1.8^c^
*Xanthorrhoea preissii*	1.0 (0.14)^cd^	0.08 (0.02)^a^	0.5 (0.06)^c^	27.7 (2.65)^c^	1.8 (0.28)^c^
					
**Huntly**					

*Acacia alata*	1.9 (0.14)^ab^	0.1 (0.03)^ab^	1.0 (0.05)^b^	38.3 (2.5)^bc^	4.3 (0.7)^c^
*Acacia pulchella*	1.9 (0.05)^a^	0.08 (0.01)^ab^	0.9 (0.03)^b^	27.1 (0.3)^c^	5.3 (0.2)^b^
*Allocasuarina fraseriana*	1.0 (0.2)^ef^	0.04 (0.01)^c^	1.0 (0.08)^b^	40.6 (0.3)^b^	8.3 (0.8)^a^
*Cyathochaeta avenacea*	1.7 (0.2)^b^	0.07 (0)^b^	0.9 (0.08)^b^	44.4 (2.1)^a^	2.1 (0.2)^d^
*Mirbelia dilatata*	1.5 (0.08)^bc^	0.07 (0.01)^b^	0.9 (0.3)^b^	32.9 (0.6)^b^	0.2 (0)^e^
*Austrodanthonia caespitosa*	0.9 (0.02)^f^	0.05 (0.01)^c^	0.7 (0.02)^c^	39.8 (2.5)^b^	0.2 (0.05)^e^
*Sphaerolobium vimineum*	1.5 (0.04)^cd^	0.06 (0)^c^	1.6 (0.04)^a^	39.7 (1.0)^b^	0.6 (0.1)^e^
*Xanthorrhoea gracilis*	1.1 (0.09)^e^	0.1 (0.01)^a^	0.7 (0.01)^c^	26.7 (0.6)^c^	1.5 (0.02)^d^
*Xanthorrhoea preissii*	0.9 (0.05)^f^	0.06 (0.01)^c^	0.6 (0.08)^c^	23.3 (0.6)^d^	1.8 (0.8)^d^
					
**Boddington**					

*Acacia alata*	1.5 (0.5)^b^	0.07 (0.0)^c^	0.8 (0.04)^b^	38.1 (1.2)^b^	6.0 (0.2)^b^
*Acacia pulchella*	1.6 (0.02)^b^	0.05 (0.1)^d^	0.8 (0.04)^b^	32.2 (1.6)^d^	5.8 (0.04)^b^
*Allocasuarina fraseriana*	0.8 (0.04)^d^	0.17 (0.02)^a^	0.8 (0.05)^b^	42.7 (0.3)^a^	8.8 (0.3)^a^
*Mirbelia dilatata*	1.7 (0.05)^a^	0.10 (0.01)^b^	1.0 (0.1)^a^	31.0 (1.2)^d^	0.4 (0.2)^e^
*Austrodanthonia caespitosa*	1.0 (0.2)^c^	0.07 (0.01)^c^	0.7 (0.03)^c^	35.3 (0.3)^c^	0.5 (0.2)^e^
*Xanthorrhoea gracilis*	0.9 (0.1)^c^	0.10 (0.02)^b^	0.6 (0.01)^d^	33.8 (0)^d^	1.4 (0)^d^
*Xanthorrhoea preissii*	0.9 (0.1)^c^	0.10 (0)^b^	0.6 (0.03)^d^	26.8 (1.3)^e^	2.0 (0.08)^c^

Tannins and ADF were similar for each location (Table 3). Water content was lower at Whiteman, while leaf area, LMA, initial heights and area of youngest fully extended leaf (YFEL) varied by location and species. Within locations (Table 6), plants at Whiteman were uniformly low in levels of phosphorus. Nutrients varied by species at all other locations. *Acacia *species were highest in nitrogen content at all locations, while *Austrodanthonia caespitosa *and *Xanthorrhoea *spp. had lowest concentration. *Allocasuarina fraseriana *had the highest levels of ADF, while the *Xanthorrhoea *species had lowest levels. Tannins were highest in *A. fraseriana *and lowest in *Xanthorrhoea *species.

**Figure 2:**
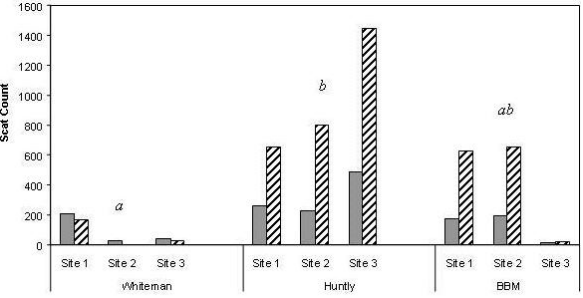
**Principal components analysis for nine plant species exposed to mammal herbivores at three locations**. iHeight = pre-exclosure removal height; iMass = pre-exclosure removal biomass (g), impact = post harvest biomass (1 - *P*) – see Methods. a = Whiteman Park, b = Huntly, c = BBM. Names for species are shown in italics.

PCA showed the likelihood of a species being browsed (% eaten) was related to impact (1 - *P *value) for the post mined systems, *r*(9) = 0.879, *P *< 0.001 at Huntly and *r*(9) = 0.846, *P *< 0.005 at BBM (Fig 2). Pearson's correlation revealed no relationship between impact and % eaten at Whiteman *r*(9) = 0.241, *P *> 0.5. There were very strong linear relations at Huntly *r*(9) = 0.879, *P *< 0.001 and BBM *r*(9) = 0.846, *P *< 0.005. Initial (pre-exclosure removal) height and initial biomass were inversely correlated with likelihood of being browsed in all systems. PCA showed phosphorus was more likely a selection candidate at BBM and Whiteman; it was not favoured at Huntly (where it was more prevalent). Leaf water content waspositively correlated with browsing in all systems. Tannins were negatively correlated in all systems.

## Discussion

Herbivory, rather than seedling competition, was the limiting factor for plant performance among post-fire (Whiteman Park) and post-mined reclamation areas. The plants in the post-fire area were far smaller and more limited in water and nutrient content than plants from the fertilised minesites. These differences may account for the dramatic increase in kangaroo density on the mines, and the decrease at Whiteman Park. High herbivory levels in the post-fire area were not expected under the theoretical framework of kangaroos selecting greater nutrient (especially nitrogen) content of plants [12,19]. Our results suggest the similar herbivory rate at Whiteman Park, despite fewer herbivores, may have been related to the inability of the post-fire system to sustain a similar level of herbivores at these sites. This is in contrast to Caughley et al [16] who suggests that herbivore numbers did not increase following fire in Mallee Scrub because plentiful winter rains left alternative food sources available elsewhere. Consequently 'burning for grazing' was recommended when winter brings plentiful rains. On both mines, it is likely that animals were increasingly attracted to the new vegetation as seedlings became more apparent, and moved onto sites from nearby forest areas.

Location effects were expected for all species; we observed early in the project that the post-mined communities were recovering far more rapidly than the post-fire community. This may be attributed to the increased water availability on the mines while the leached sands of Whiteman Park has resulted in plants showing lower levels of phosphorus, potassium and usually nitrogen. We expected a higher level of interactions between herbivory and plant competition. Gurevitch [33] and Fowler [34] suggested that the removal of competitors has a greater effect in the absence of herbivores. This was not so for the nine species examined here: increased density (competition) made little difference to performance in the presence or absence of herbivores.

Impact was a function of the level of herbivory on the minesites, but not post-fire sites. On the minesites, the stronger relation between browsing and impact (biomass loss) may be due to the increased kangaroo density and the high plant vigour from fertilised seedlings [11]; when plants were consumed they were eaten to death. The non significant linear correlation between browsing and impact at Whiteman Park may have been due to mixed diet factors and plant apparency [35]. When nutrients are limited herbivores will often take selectively from plants and plant parts without heavy consumption of each individual [17]. Small grasslike plants at Whiteman are normally favoured, the Xanthorrhoea species may have been browsed less intently because they were not as apparent to the herbivores as larger species.

It is interesting to note that at the driest location, Whiteman Park, leaf water content was most closely correlated with extent of browsing. The same trend occurred with phosphorus and potassium, as they both became criteria for selection when in short supply. This is consistent with the findings of Villalba et al. [6] and Scott and Provenza [36] for bovines and Parsons [5] for macropods. Each has demonstrated that the criteria for herbivore selection may change according to availability within the diet.

Among the nine species we examined, the selection (% browsed) of a plant species was inversely correlated with initial (pre-exclosure removal) shoot mass. This seems to be due to smaller species having a grasslike growth form which is more attractive to kangaroos. However, we caution that this is not always the case. When working with 24 species from the same system, we have demonstrated that some commonly browsed species had the highest biomass when browsed, though in the case of one shrub, *Viminaria juncea*, its foliage was also grasslike [21].

## Authors' contributions

MP carried out all experimental studies within the post-mining area and drafted the manuscript. CR performed all post-fire studies. BL assisted research in post-mining and post-fire and assisted all drafts of the manuscript. KD provided all chemical analyses and assisted the first draft of the manuscript. MF performed physical measurements in the field and laboratory for post-fire and post-mining studies. All authors have read and approved the final manuscript.
